# An RNAi-Based Approach to Down-Regulate a Gene Family *In Vivo*

**DOI:** 10.1371/journal.pone.0080312

**Published:** 2013-11-12

**Authors:** Jeehee Kim, Aurora Badaloni, Torsten Willert, Ursula Zimber-Strobl, Ralf Kühn, Wolfgang Wurst, Matthias Kieslinger

**Affiliations:** 1 Institute of Clinical Molecular Biology and Tumor Genetics, Helmholtz Zentrum München, German Research Center for Environmental Health, Munich, Germany; 2 Division of Neuroscience, San Raffaele Scientific Institute, Milan, Italy; 3 Department of Gene Vectors, Helmholtz Zentrum München, German Research Center for Environmental Health, Munich, Germany; 4 Institute of Developmental Genetics, Helmholtz Zentrum München, German Research Center for Environmental Health, Munich, Germany; University of Frankfurt - University Hospital Frankfurt, Germany

## Abstract

Genetic redundancy poses a major problem to the analysis of gene function. RNA interference allows the down-regulation of several genes simultaneously, offering the possibility to overcome genetic redundancy, something not easily achieved with traditional genetic approaches. Previously we have used a polycistronic miR155-based framework to knockdown expression of three genes of the early B cell factor family in cultured cells. Here we develop the system further by generating transgenic mice expressing the RNAi construct *in vivo* in an inducible manner. Expression of the transgene from the strong CAG promoter is compatible with a normal function of the basal miRNA/RNAi machinery, and the miR155 framework readily allows inducible expression from the *Rosa26* locus as shown by Gfp. However, expression of the transgene in hematopoietic cells does not lead to changes in B cell development and neuronal expression does not affect cerebellar architecture as predicted from genetic deletion studies. Protein as well as mRNA levels generated from *Ebf* genes in hetero- and homozygous animals are comparable to wild-type levels. A likely explanation for the discrepancy in the effectiveness of the RNAi construct between cultured cells and transgenic animals lies in the efficiency of the sequences used, possibly together with the complexity of the transgene. Since new approaches allow to overcome efficiency problems of RNAi sequences, the data lay the foundation for future work on the simultaneous knockdown of several genes *in vivo*.

## Introduction

One of the surprising findings of gene targeting in mice are knock-out animals with no obvious phenotype. While a proportion of these cases is likely due to incomplete phenotypic testing or a manifestation only under certain non-standard conditions, it is estimated that 10 - 15% of knock-out mice do not have an obvious phenotype [1]. The vast majority of such cases is thought to derive from functional redundancy between duplicated and highly homologous genes [2]. In favour of this hypothesis, duplicate genes occur in all organisms, especially in multicellular eukaryotes with approximately 50% of mouse genes having a related member in the genome [3–5]. While these facts have interesting and sometimes hard to reconcile implications for evolutionary biology [6,7], gene duplications and redundancy often mask the true contribution of single genes, making it difficult to discern the function of each copy by knocking out individual genes [8]. 

Interestingly, conserved gene duplicates in vertebrates are specifically enriched for signal transduction and developmental function [9]. Good examples of this are the MRF family of transcription factors, acting as master regulators of vertebrate skeletal muscle development, and the Hox gene cluster [10]. Another interesting case is the early B cell factor (Ebf) family of transcription factors. All four members show a high level of sequence conservation, reaching 80 - 90% at the nucleotide level within conserved domains, and all four members bind to the same DNA sequence [11,12]. Despite a strong expression in tissues like the olfactory epithelium and in many neuronal cells, deletion of *Ebf1* results in a distinct phenotype, i.e. the complete block of early B cell development [13]. This finding was unexpected, but Ebf1 is the only family member expressed in hematopoietic cells, whereas the tissues mentioned above express other Ebf genes in a largely overlapping manner [14–17]. In fact, in addition to B cells, Ebf1 is the only Ebf gene expressed in the embryonic striatum and in cranial nerve nuclei, and *Ebf1*-deficient mice display atrophy and abnormal cellular migration, axonal fasciculation and projection [18–20]. *Ebf2* and *Ebf3* are also expressed in a largely overlapping pattern, and deletion of the single genes leads to a mixture of distinct and overlapping phenotypes causing lethality at the age of approximately two months or immediately after birth respectively. *Ebf2* null mice display abnormalities in the cerebellum and peripheral nerve [21,22]. Mice double heterozygous for *Ebf2* and *Ebf3* recapitulate common defects, such as defective projection of olfactory neurons, but do not display some of the defects occurring only in single knock-outs, arguing that the function of *Ebf* genes is dose-dependent and at least partially redundant [23]. 

In an effort to overcome functional redundancy and examine the contribution of Ebf factors in general to the support of hematopoietic stem cells, we employed the SIBR (synthetic inhibitory BIC-derived RNA) vectors, a new approach to down-regulate several genes simultaneously based on RNA interference [24]. In this system shRNA sequences are embedded into the framework of miR155/BIC including the flanking sequences, which are cleaved by the RNAseIII enzyme Drosha. This allows the concatemerisation of several shRNAs, and the inclusion of a marker gene, in our case *Gfp*. Furthermore, regulated expression of the RNAi construct is possible since it uses RNA polymerase II-driven promoters. Overall, this results a flexible system to express several shRNAs together with a marker gene in a single polycistronic mRNA driven by PolII [25]. We were able to successfully down-regulate Ebf1, Ebf2 and Ebf3 and to demonstrate redundancy between these genes in retrovirally infected cultures of osteoblastic cells [24]. The same vector has been used also by other groups to inhibit the Ebf proteins in a variety of different cells [26,27]. As this system proved useful in cell culture approaches in different contexts, we wanted to develop it further and overcome the limitations of *in vitro* approaches and retroviral infections. Here, we report the generation of transgenic mice with a targeted insertion of the SIBR-based *EbfRNAi* construct into the murine *Rosa26* locus, and describe the effects of its induced expression.

## Results

### Generation of EbfmiRNA transgenic mice

The successful down-regulation of Ebf1, Ebf2 and Ebf3 using one miRNA construct allowed us to analyse the biological effects of this highly conserved protein family in cell culture [24]. While this approach opens new possibilities in studying biological roles independent of redundancy, it remains limited to *in vitro* assays and the expression of the miRNA construct via transfection or viral infection. To overcome these limitations we wanted to develop this system further and use it *in vivo* by generating mice with a targeted insertion. We had previously generated two different miRNA constructs, RNAi-a and RNAi-b, targeting the mRNAs of *Ebf1*, *Ebf2* and *Ebf3* at different positions, primarily to control for off-target effects of the miRNA generated [24,28]. The single shRNAs bind to regions of the Ebf mRNAs corresponding to the DNA binding or the adjacent IPT/TIG-domain (of unknown function) of the encoded proteins (Figure S1A) and are concatemerised as indicated (Figure S1B). Therefore, as two different constructs were available, we wanted to analyse the effectiveness of the RNAi constructs in detail before going *in vivo*. Therefore, HEK293T cells were transfected with expression plasmids for flag-tagged Ebf1, Ebf2 and Ebf3. In parallel, all six single constructs (two per *Ebf* gene), double and triple constructs were co-transfected. Co-expression of the single RNAi constructs resulted in the down-regulation of the respective Ebf protein, but left the other family members relatively unaffected, indicating specificity but also a high level of effectiveness. The double RNAi constructs against Ebf2 and Ebf3 led to their strong down-regulation, but left Ebf1 relatively unchanged, demonstrating that neither specificity nor effectiveness are compromised by the concatemerisation via the flanking region of miR155. Finally, expression of the triple RNAi constructs induced a loss of Ebf proteins to almost undetectable levels (Figure S2). Although the single RNAi constructs seemed to show slight differences in down-regulation efficiencies, these were minor and could be due to experimental variances. In fact, no differences were observed with the double and triple constructs, confirming data from the down-regulation of endogenous Ebf proteins [24]. Therefore, we decided to use both constructs for the generation of targeted mouse ES cells.

**Figure 1:**
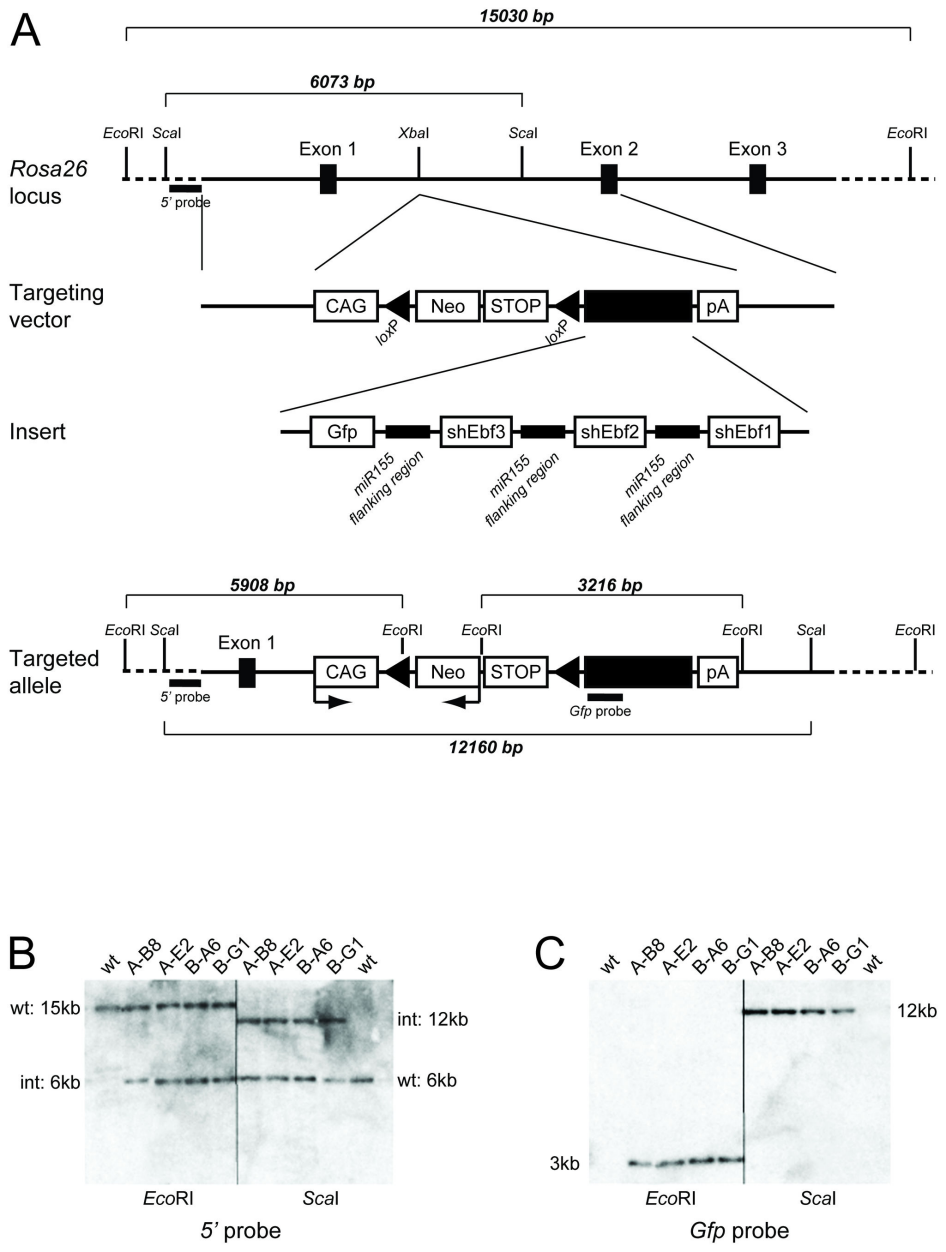
Generation of *EbfmiRNA* transgenic murine ES cells. (A) Schematic representation of the *Rosa26* wild-type locus. A solid line represents regions involved in the targeting construct, while a dashed line depicts genomic regions beyond the targeting construct. The targeting strategy consists of inserting the transgene into the XbaI site in intron 1, which is flanked by homology arms including exons1, 2 and 3. The transgene consists of the synthetic CAG-promoter, a *PGK::Neo* cassette with a transcriptional Stop sequence flanked by *loxP* sites, the RNAi insert as a black box, and a polyA sequence. In the third line the insert is detailed as consisting of the *Gfp* gene, and DNA sequences encoding for short hairpin mRNA against *Ebf3*, *Ebf2* and *Ebf1*. These sequences are surrounded by the flanking region of miR155, which is recognised by the ribonuclease Drosha. The locus after recombination is depicted in line 4, also indicating the direction of transcription from the CAG promoter and the *PGK::Neo-Stop* cassette. Endogenous and newly introduced restriction sites are indicated and the resulting fragment lengths for wild-type and targeted allele with specific restriction enzymes are given. Southern blot probes corresponding to the *5*’ region and to the *Gfp* gene are indicated. (B) Southern blot of genomic DNA from one wild-type and four mutant ES cell clones digested with *Eco*RI and *Sca*I as indicated, and hybridised to the *5*´ probe (as shown in A). Bands corresponding to the wild-type (wt) and the targeted (int) allele are indicated. (C) Southern blot of genomic DNA as in B, hybridised to the internal *Gfp* probe. Bands of the correct size appear in all four targeted ES cell clones as indicated.

Since miRNAs are standard class II mRNAs driven by RNA polymerase II [29], yet depend on strong expression, we decided to target the insertion to the *Rosa26* locus. However, instead of relying on the endogenous transcriptional activity of the locus we decided to use the synthetic CAG (CMV early enhancer/ chicken β-actin) promoter, which has very strong transcriptional activity [30,31]. In combination with a transcriptional Stop site and a *PGK::Neo* resistance cassette, both flanked by *loxP* sites, immediately downstream of the CAG promoter (Figure 1A), this construct allows strong and tissue-specific expression of the insert [32]. The insert, which follows immediately after the *loxP* sites consists of the *Gfp* gene and the short hairpin sequences concatemerised by the flanking region of miR155 (Figure 1A). This sequence was cut from the pcDNA6.2-GW/EmGFP vector that was described before [24], and is also analysed here (Figure S2).

The targeting construct was used for electroporation of IDG3.2 murine ES cells, which were selected by G418. 600 resistant ES cell clones were picked and analysed for correct integration of the construct by Southern blot. In case of the external *5'* probe, the appearance of a 6kb band in an *EcoR*I digestion and of a 12 kB band with a *Sca*I digestion indicates the correct integration of the construct. Figure 1B shows two clones with integration of the RNAi-A (A-xx) and two clones with integration of the B construct (B-xx). Hybridising Southern Blot to the internal *Gfp* probe results in the predicted bands of 3 kb for the *EcoR*I digest and 12 kb for the *Sca*I digest, confirming the correct and unique integration of the targeting vector (Figure 1C). In addition to the Southern Blot, all positive clones were also confirmed by sequencing. Overall, a 7.5% targeting efficiency was achieved over 600 clones tested, confirming the high recombination efficiency published for this locus [33,34].

### No leakiness and high inducibility of the transgene in murine ES cells

**Figure 2:**
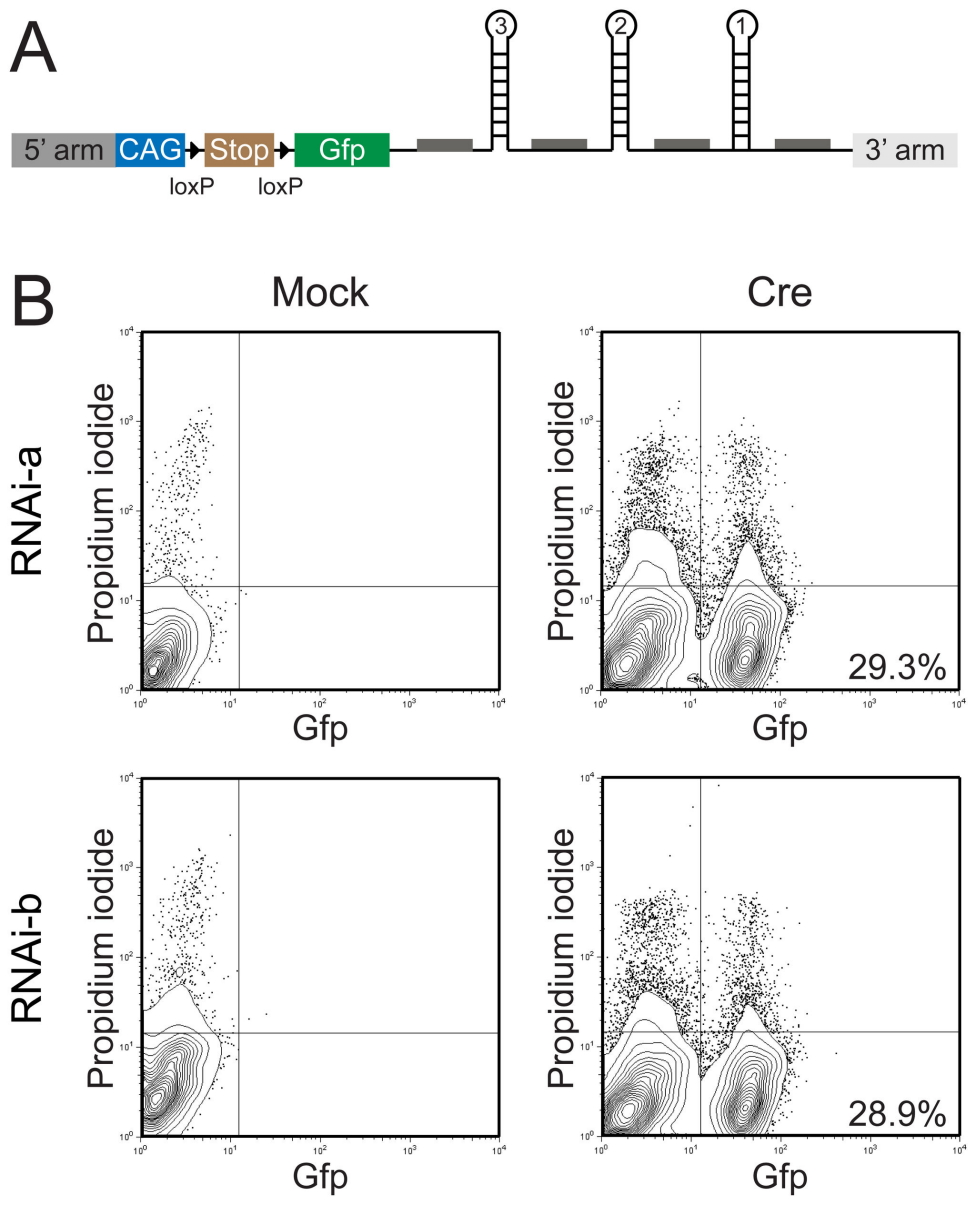
The transgene shows no leakiness and high inducibility in murine ES cells. (A) Schematic depiction of the transgene inserted into the *Rosa26* locus. Expression is driven by the strong CAG promoter, but aborted due to the presence of the transcriptional Stop sequence. Upon Cre mediated deletion, the stop cassette is removed and a polycistronic mRNA encoding for Gfp and short-hairpin sequences against *Ebf3*, *Ebf2* and *Ebf1* gets expressed. (B) ES cell clones tested by Southern blot as correctly targeted for either the RNAi-a or RNAi-b construct were transfected with the pGK-Cre-bpA plasmid encoding for the Cre recombinase or with the empty parental vector (mock). 48 h after transfection, cells were analysed by FACS for the expression of Gfp and for cell death by propidium iodide. Cells have been gated for FSC/SSC.

To test the transgene functionally before going *in vivo*, we transfected ES cell clones with the pGK-cre-bpA plasmid (kind gift of Dr. Kurt Fellenberg, Institute of Genetics, University of Cologne), which leads to the expression of the Cre-recombinase in transfected cells. In consequence, the transcriptional Stop sequence should be deleted and the transgene including the *Gfp* gene should come under control of the CAG promoter (Figure 2A). ES cells were harvested 48h post-transfection and analysed by flow cytometry for the expression of Gfp. Figure 2B indicates that cells transfected with the mock vector do not express *Gfp*, and a low percentage of cells shows apoptosis as detected by propidium iodide. Transfection of the Cre-encoding plasmid however, leads to an expression of *Gfp* in approximately 30% of cells, again with a low percentage of cells showing apoptosis (Figure 2B). This indicates that the construct is not leaky, i.e. there is no expression of the transgene in presence of the Stop sequence, and that the transgene is efficiently induced via Cre-mediated excision of the Stop cassette. Furthermore, expression of the transgene does not influence viability of the cells. 

### No leakiness, high inducibility and strong expression of the transgene *in vivo*

Since all tests in ES cells provided evidence for the correct insertion and the functioning of the transgene, we proceeded to generate transgenic mice by standard conditions. Two different ES cell clones for each RNAi construct were injected into blastocysts and chimeric mice were obtained with both transgenic constructs, RNAi-a and RNAi-b. However, germ-line transmission was only successful with chimeric mice transgenic for the RNAi-a construct. As RNAi-a and RNAi-b did not differ significantly in their ability to down-regulate Ebf proteins *in vitro* (Figure S2 and [24]), we continued to analyse these mice and will refer to them as *Rosa26* ^*RNAi*^. 

**Figure 3:**
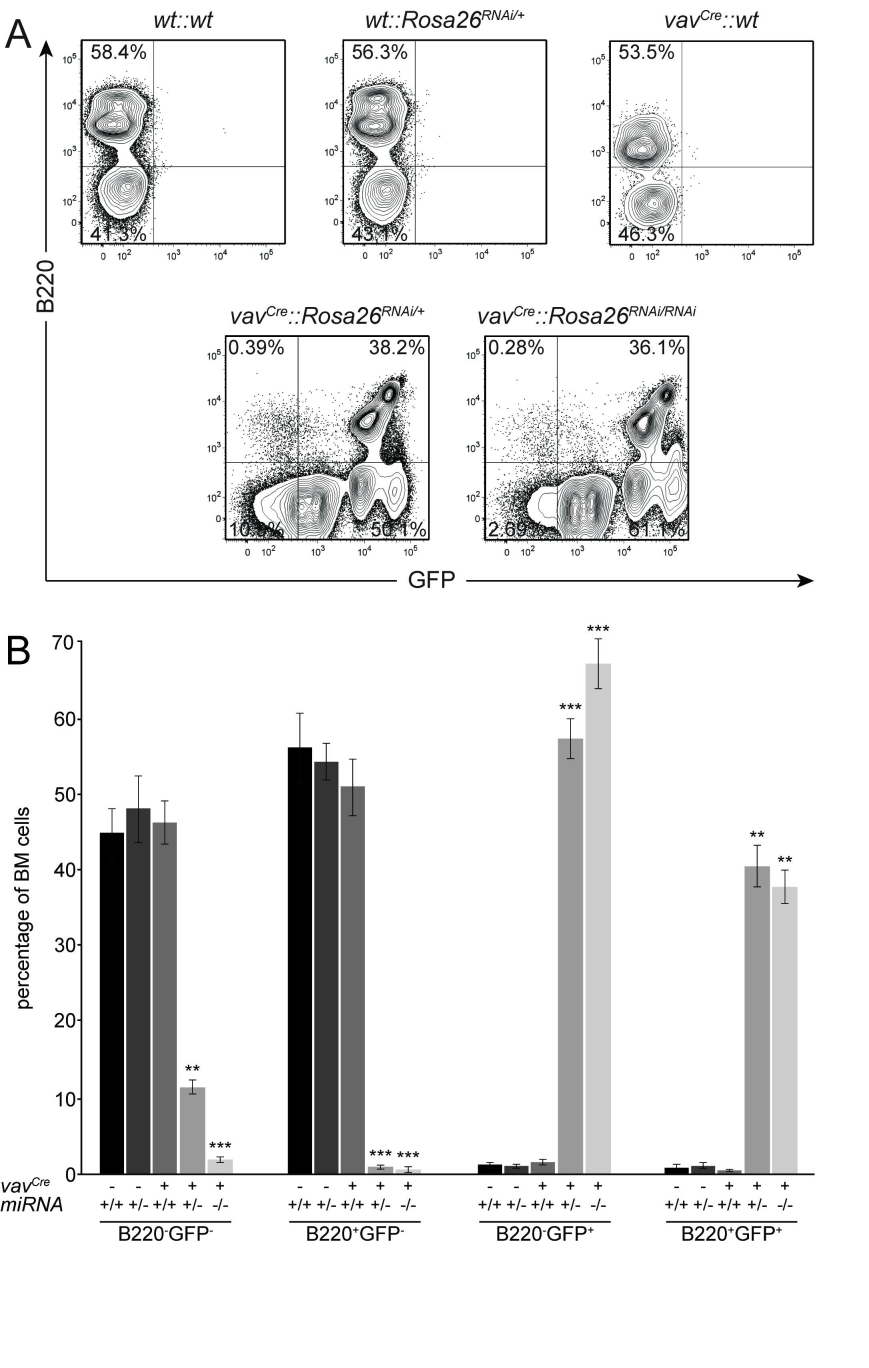
No leakiness, high inducibility and strong expression of the transgene *in* *vivo*. (A) Single cell suspensions from bone marrow of mice with the indicated genotypes were analysed for the expression of Gfp and the B cell marker B220. Gfp-positive cells are observed only in combination of the *vav* ^*Cre*^ and the transgenic *Rosa26* ^*RNAi*^ genotypes. All other genotypes show expression of B220, but a lack of Gfp. Note the slight shift in expression of Gfp from *Rosa26* ^*RNAi/+*^ to *Rosa26* ^*RNAi/RNAi*^, due to the presence of two alleles of the transgene. Cell have been gated for FSC/SSC and as PI^-^. (B) Statistical analysis of bone marrow cells stained and analysed as in A. Significant differences based on genotypes are observed only in combination of the *vav* ^*Cre*^ and *Rosa26* ^*RNAi*^ transgenes compared to all other genotypes; n=3-4; error bars=SD; ** p<0.01, *** p<0.001.

To determine leakiness, inducibility and functionality *in vivo*, we wanted to induce expression of the transgene in hematopoietic cells. Ebf1 is expressed in early B cells and is necessary for the transition from fraction A to fraction B during B cell development [13]. Furthermore, miRNAs and the protein machinery required for their processing are important for the development of other hematopoietic cells like T and B cells [35]. Thereby, the functionality of the transgene can be tested in early B cells and potentially unspecific or off-target effects by examining T cell development, making the hematopoietic system ideal to study both aspects. To this end, we crossed the *Rosa26* ^*RNAi*^ mouse line to the *vav* ^*Cre*^ line, which is active in hematopoietic stem cells and their derivatives [36]. In a first analysis, we wanted to determine the inducibility and the level of expression of the transgene, as these are two critical parameters for the effectiveness of RNAi. Single cell suspension from the bone marrow of adult mice from various genotypes was analysed by flow cytometry for the expression of *Gfp*. In all control mice, *wt::wt*, *wt::Rosa26* ^*RNAi/+*^ and *vav* ^*Cre*^ *::wt*, no expression of *Gfp* and a percentage of approximately 50% B220-positive cells could be detected, indicating again no leakiness of the construct. A small reduction in the number of B220-positive cells was observed in the presence of the recombinase, but this phenotype did not reach statistical significance, therefore it might represent a slight tendency, but does not proof phenotypic changes due to the expression of the Cre recombinase. The vast majority of bone marrow from *vav* ^*Cre*^ *::Rosa26* ^*RNAi/+*^ is shifted into the Gfp positive channel, with virtually all B220-positive cells also expressing *Gfp*. The level of expression of Gfp varies between several different populations, but ranges between one to three shifts in logarithmic units above wild-type, with the majority of cells at around two log-shifts (Figure 3A). Bone marrow cells from *vav* ^*Cre*^ *::Rosa26* ^*RNAi/RNAi*^ show exactly the same pattern of Gfp-positive cells when plotted against B220, but the expression of *Gfp* is shifted to even higher levels, with very few cells remaining negative for Gfp (Figure 3A). Statistical analysis shows that the described differences in the expression of *Gfp* are highly significant, and also indicate that the number of B220-positive cells is reduced from 53.8% in wild-type animals to 40.4% in *vav* ^*Cre*^ *::Rosa26* ^*RNAi/+*^ and 38.6% in *vav* ^*Cre*^ *::Rosa26* ^*RNAi/RNAi*^ mice, indicating a potential but weak influence of the transgene on B cell development (Figure 3B).

### No alterations in early B cell fractions mediated by expression of the transgene

**Figure 4:**
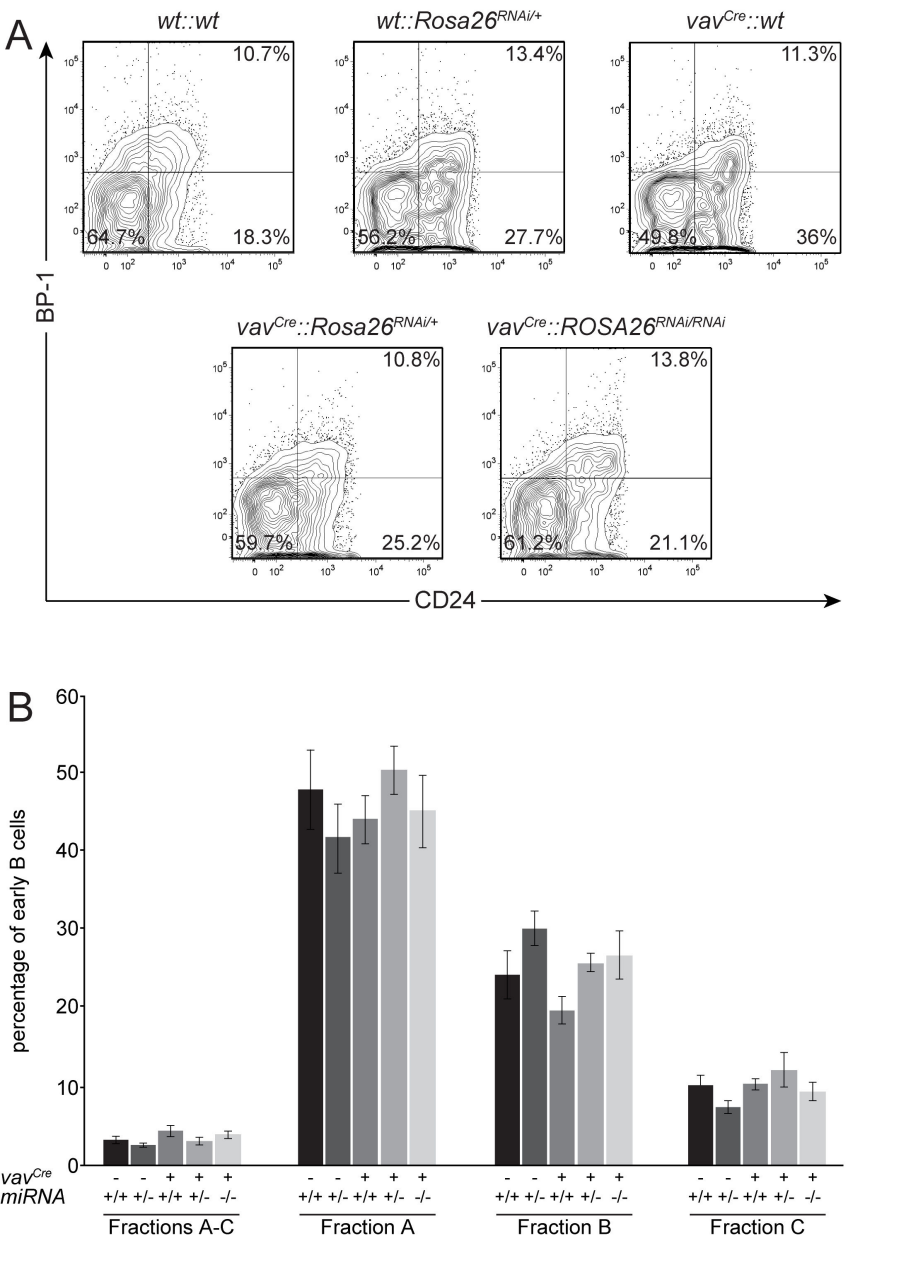
No alterations in early B cell fractions mediated by expression of the transgene. (A) Single cell suspensions from bone marrow of mice with the indicated genotypes were analysed for the percentage of early B cell fractions. Cells were stained with propidium iodide, B220, CD43, BP-1, HSA and CD24. Propidium iodide negative cells are gated as B220/CD43 double positive (see Figure S3), and analysed for expression of HSA/CD24 allowing to distinguish fractions A (B220^+^CD43^+^CD24^-^BP-1^-^), B (B220^+^CD43^+^CD24^+^BP-1^-^) and C (B220^+^CD43^+^CD24^+^BP-1^+^) of B cell development. Representative examples of the indicated genotypes are shown. Cells were further gated for FSC/SSC. (B) Statistical analysis of bone marrow cells stained and analysed as in A. No significant differences based on genotypes are observed; n=3-4; error bars=SD.

As we detected a high expression of the transgene, particularly in B cells and a slight reduction of B220-positive cells, we continued to analyse the functionality of the RNAi transgene. Since Ebf1 is the only Ebf family member expressed in haematopoietic cells and is required for the progression from fraction A to fraction B in early B cell development [13], we expected a block or at least a down-regulation of cells in the transition from fraction A to fraction B, depending on the efficiency of the construct. Single cell suspensions from mice of the indicated genotypes were stained for B220 and CD43, and double positive cells, which comprise fractions A - C were analysed. No differences in the percentage of these cells were detected (Figure S3). For further analysis, these cells were gated and analysed for expression of BP-1 and CD24, allowing the individual fractions to be distinguished. Again, no differences beyond standard variations in fractions A (BP-1^-^CD24^-^), B (BP-1^-^CD24^+^), or C (BP-1^+^CD24^+^) could be detected in the various genotypes (Figure 4A). Statistical analysis confirms this for all fractions tested. Neither in the B220^+^CD43^+^ Fractions A - C, nor in the individual fractions is it possible to detect any significant changes (Figure 4B). 

### Expression of the transgene does not interfere with Ebf1 levels *in vivo*

**Figure 5:**
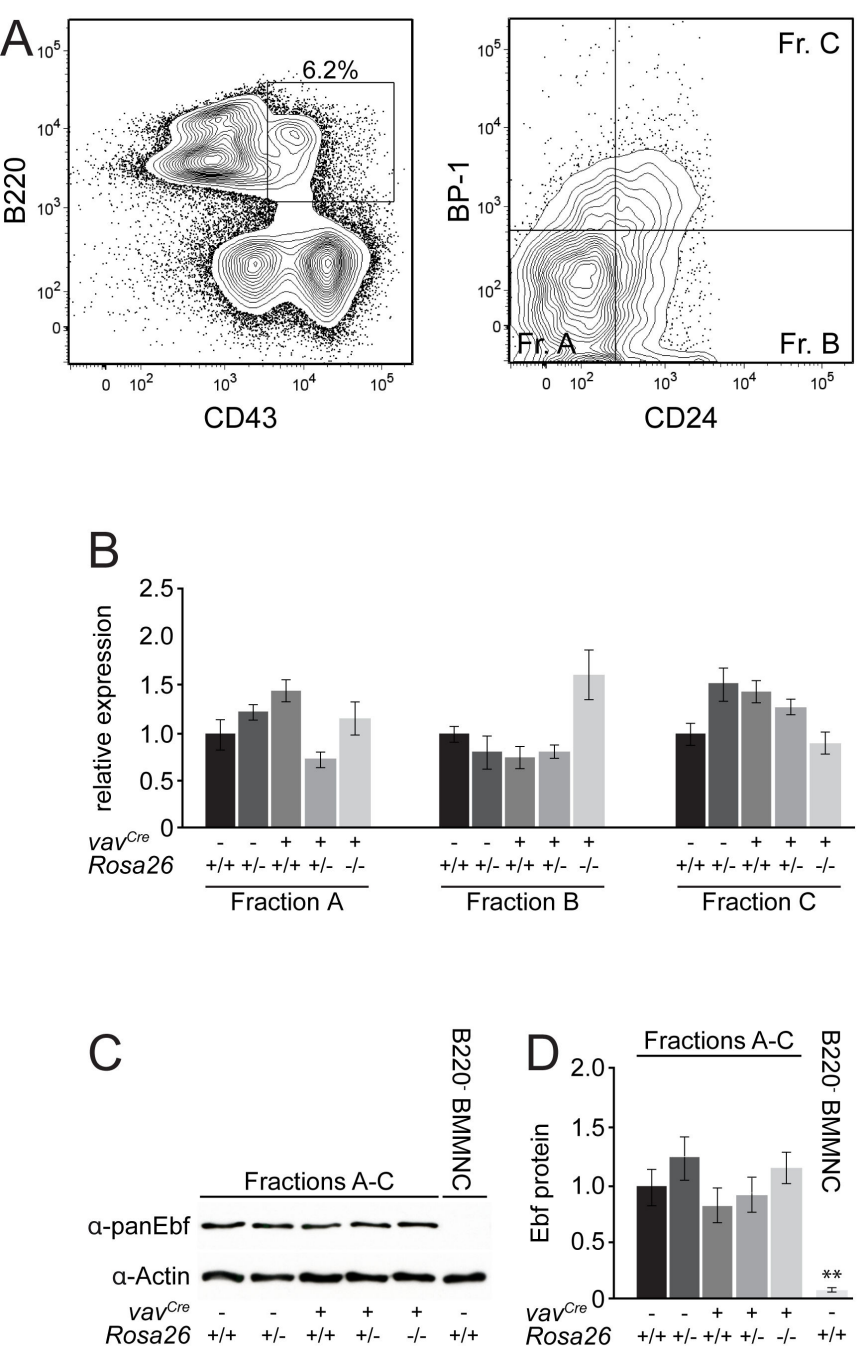
Expression of the transgene does not interfere with Ebf1 levels *in* *vivo*. (A) Single cell suspensions from bone marrow of mice with the same genotypes as before were prepared and stained for B220/CD43 and CD24/BP1. Cells were gated in flow cytometry for FSC/SSC and as B220^+^CD43^+^ as indicated in the left panel in a representative example. The right panel shows the gates used for sorting of cells from fraction A (B220^+^CD43^+^CD24^-^BP-1^-^), B (B220^+^CD43^+^CD24^+^BP-1^-^) and C (B220^+^CD43^+^CD24^+^BP-1^+^). These B cell fractions were sorted from all the genotypes used throughout this study. (B) Sorted cells from different B cell fractions of the indicated genotypes were subjected to qPCR analysis of Ebf1. All values were normalised to *HPRT* and the expression of Ebf1 in wild-type cells was set to 1 in each case; n=3-4; error bars=SD. (C) B220^+^CD43^+^ cells were sorted from total bone marrow of mice with the indicated genotypes and used for analysis of expression of Ebf1 by Western blot (Fractions A-C). Bone marrow from wild-type mice was depleted of B220^+^ cells (B220^-^ BMMNC) and used as negative control, β-actin is used as loading control. (D) Measurement of the amount of protein present in the B cell fractions and control as depicted in C using ImageJ; n=3-4; error bars=SD, ** p<0.01.

As no differences in early B cell fractions could be detected in hematopoietic cells, yet the cells express the transgene, we wanted to determine the levels of Ebf1 under these conditions. Therefore, we sorted fractions A, B and C according to the sorting gates indicated in Figure 5 A from mice carrying the same genotypes as before. Analysis of these early B cell fractions for the expression of *Ebf1* revealed no significant differences in the amount of mRNA even in *vav* ^*Cre*^ *::Rosa26* ^*RNAi/+*^ versus *vav* ^*Cre*^:*: Rosa26* ^*RNAi/RNAi*^ mice (Fig. 5B). Since RNAi can also inhibit protein translation, we tested the protein levels of early B cells. As the number of cells after fractionation is too low to allow for testing of individual fractions, we sorted B220^+^CD43^+^ fractions A - C, and subjected those to Western blot analysis. Using a pan Ebf antibody recognising Ebf1, Ebf2 and Ebf3, we could detect comparable amounts of Ebf protein in cells from all genotypes. For control, bone marrow mononuclear cells were depleted of B220^+^ cells and included in the assay. No band was detected in these cells, confirming that no Ebf protein is expressed in hematopoietic cells besides Ebf1 in B cells (Figure 5C). Quantification of the protein amount by densitometry confirmed this observation by showing that no significant differences in Ebf protein levels exist based on the various genotypes (Figure 5D). Therefore we conclude that although the transgene mediates down-regulation of Ebf proteins in cell culture, and it is expressed at high levels in B cells from transgenic mice, its expression does not affect Ebf1 protein levels *in vivo*.

### No alteration in cerebellar cyto-architecture in the Ebf2^GFPiCre^::Rosa26^RNAi^ line

**Figure 6:**
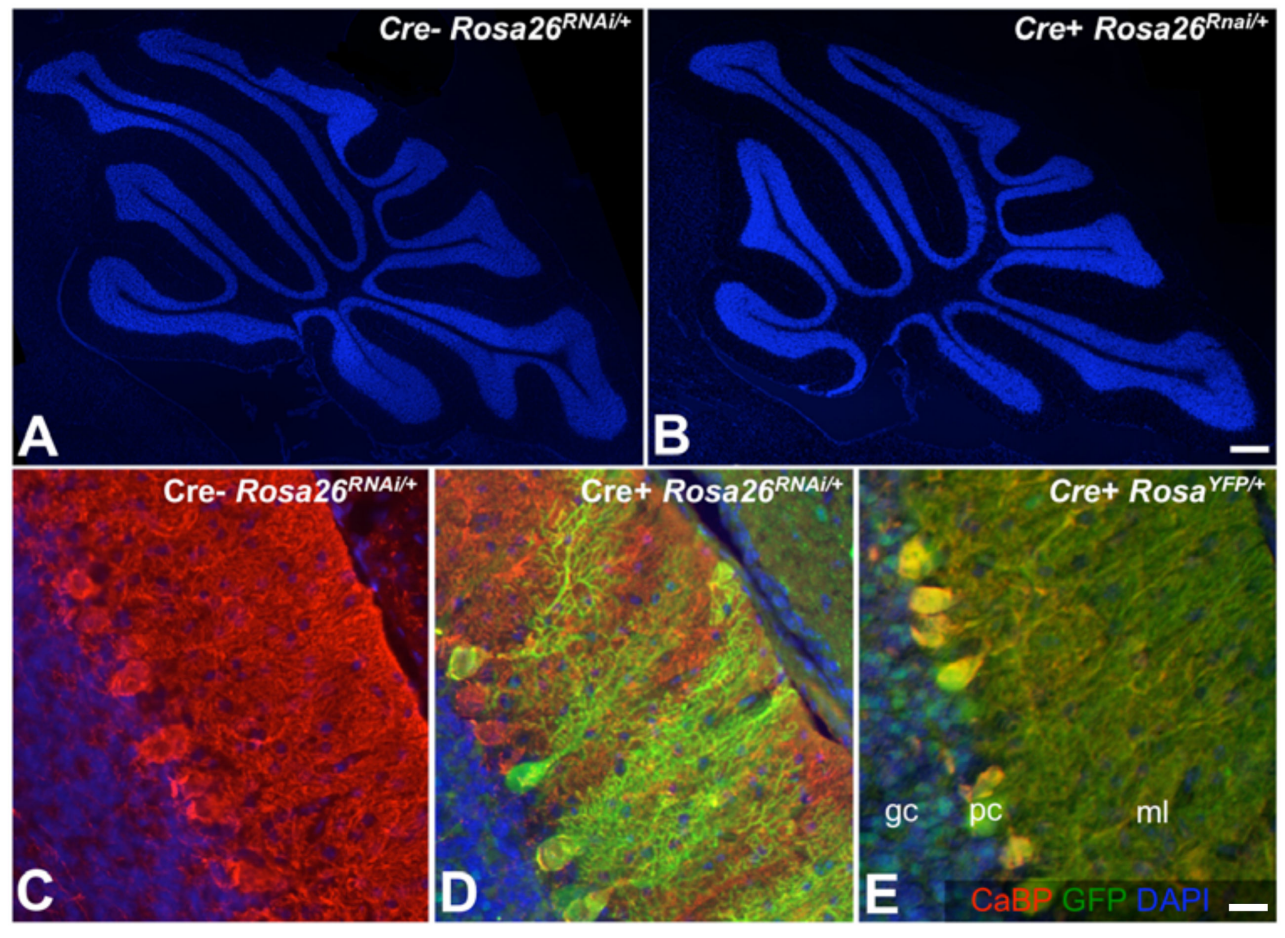
No relevant alterations in the adult cerebellum as a result of RNAi activation in the early cerebellar primordium. (A-B) Low magnification DAPI stainings of postnatal day 60 cerebella. Normal lobulation in the RNAi-expressing cerebellum (*Ebf2* ^*Cre*^::*Rosa* ^*26RNAi/+*^, panel B), compared to a Cre- control (A). (C-D) Sagittal sections of adult cerebella stained for the Purkinje cell (PC)-specific marker calbindin (CaBP, red) and for Gfp. RNAi-expressing PCs (Gfp^+^, panel D) develop normal somata and dendritic arbors positive for CaBP. Panel E (normal control) shows sagittally sectioned CaBP^+^ PCs from an *Ebf2* ^*Cre*^::*Rosa26* ^*YFP/YFP*^ mouse. Size bars: B, 200 µm; E, 25 µm.

Previous studies have clearly indicated that targeted deletion of one member of the Ebf gene family (*Ebf2*) impairs cerebellar development. In particular, cerebellar Purkinje cells (PCs) are decreased in number affecting the size and lobulation of the null cerebellum. One PC subpopulation is selectively reduced, and PC migration into the cerebellar cortex is impaired or delayed [21,37]. Ebf2 exerts an autocrine/paracrine control over PC survival by activating *Igf1* gene expression [27]. In light of these previous findings, we tested the combined effect of *Ebf1-3* down-regulation on cerebellar development. This analysis was conducted on the adult (P60) progeny of an *Ebf2* ^*Cre*^ x *Rosa26* ^*RNAi/+*^ cross. *Ebf2-Cre* is activated at the outset of PC development, and robust Cre levels are detected in the cerebellar primordium as early as E11.5 [26]. This transgene marks the PC lineage, as shown by genetic fate mapping (A.B., unpublished observations). Our results clearly indicate that *Ebf2* ^*Cre*^::*miRNA* ^*+/-*^ mice feature no major cerebellar defects (Figure 6A-D). DAPI staining indicated that cerebellar lobulation is unaffected by RNAi activation in PC progenitors. Moreover, transgenic PCs express normal levels of Calbindin (CaBP), a Ca^2+^ binding protein whose levels decrease rapidly upon PC degeneration. Finally, RNAi-expressing PCs exhibit a normally branched dendritic tree. Thus, no obvious abnormalities were scored in RNAi-expressing cerebella. 

### Expression of Gfp and the miRNA-transgene are correlated but do not influence Ebf protein levels

**Figure 7:**
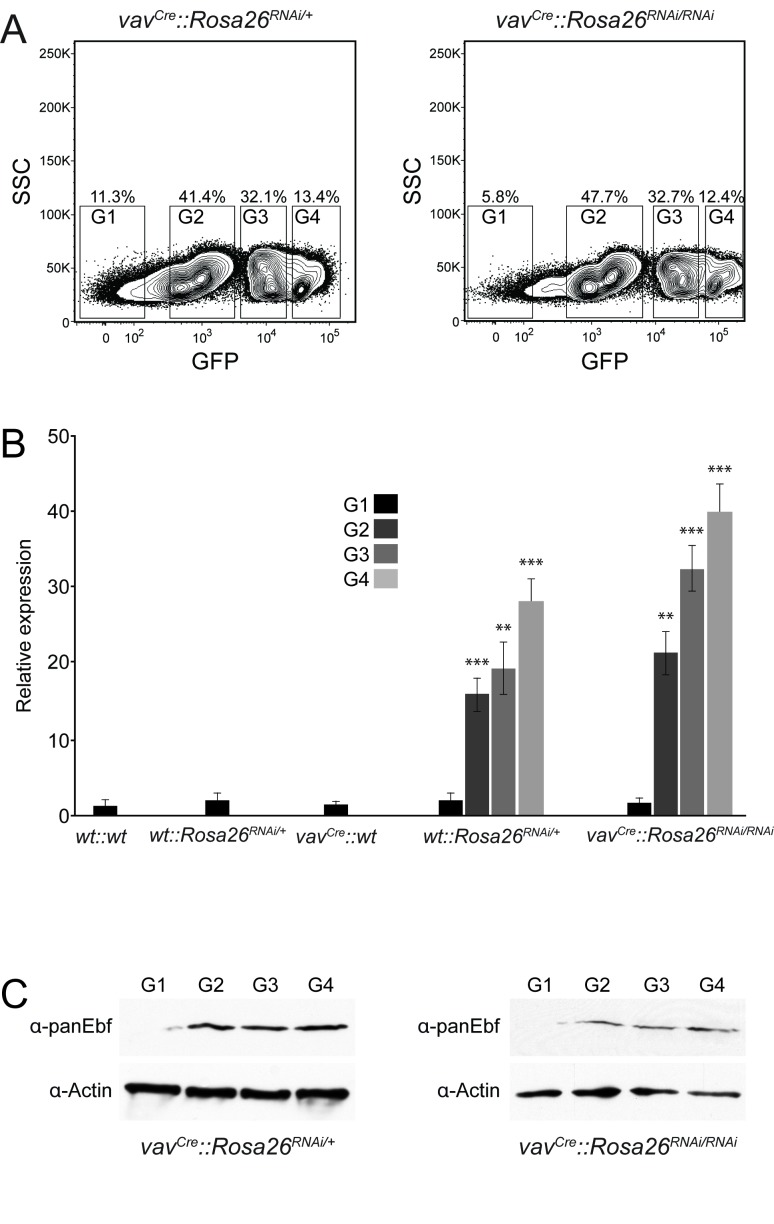
Expression levels of Gfp and transgene correlate, but do not influence Ebf protein. (A) Bone marrow cells from mice with the indicated genotypes were sorted as negative (G1), low (G2), middle (G3) and high (G4) for the expression of Gfp. (B) Cells sorted according to the gates in (A) were analysed for the expression of the miRNA by qPCR. Additionally, bone marrow was isolated from control mice as indicated and used in this analysis. n=3-4; error bars=SD, ** p<0.01, *** p<0.001. (C) Cells sorted according to the gates in (A) were analysed for the presence of Ebf1 protein by western blot. β-actin is used as loading control and the genotypes of the donor mice are indicated. For western blot, three biological replicates were pooled and analysed together.

The data indicate a clear discrepancy between the functionality of the construct *in vitro* and *in vivo*. One possible explanation is an insufficient expression level of the transgene. So far we have analysed Gfp-positive cells as one bulk population, but as shown in Figure 3A, the expression of Gfp shows a broad spectrum, from high to low expression in largely three distinct populations. This offers the possibility to analyse these populations individually, to examine if expression-related effects are masked in the bulk analysis. To this end, we isolated low, middle and high Gfp-expressing cells in addition to Gfp-negative cells by flow cytometry from *vav* ^*Cre*^ *::miRNA* ^*+/-*^ and *vav* ^*Cre*^ *::miRNA* ^*-/-*^ mice. Interestingly, the percentage of cells in the respective gates does not differ between these two genotypes, but the expression of Gfp is clearly higher in *vav* ^*Cre*^ *::miRNA* ^*-/-*^, reflecting the presence of two instead of one allele of the transgene (Figure 7A). 

The expression of the transgene in these sorted populations was analysed in qPCR by primers spanning from the 3'-end of Gfp to the last inserted miRNA sequence. As shown in Figure 7B, Gfp-negative cells from all examined genotypes displayed no detectable expression of the transgene, while cells from gates 2 - 4 show increasing expression of the transgene (Figure 7B), revealing a correlation in the expression of the transgene with Gfp. Therefore, if expression plays a role, we might be able to see an influence on Ebf1 protein levels in the most highly expression populations. However, western blot analysis of cells sorted according to the gates showed that Ebf1 was present in the different fractions at comparable levels (Figure 7C), indicating that too low expression of the transgene alone does not explain the observed inefficiency *in vivo*.

## Discussion

RNAi has evolved as a means to down-regulate the expression of single target genes *in vivo* [38]. Initially, short-hairpin RNAs were driven by RNA polymerase III-dependent promotors to affect the expression of a single target gene [39–44]. Tissue-specificity can be achieved via the disruption of the promotor by a neomycin-resistance cassette flanked by *loxP* sites [45–47]. However, neither does this system allow to incorporate a marker gene, nor to choose the activating potential of the promotor. When it was found that over-saturation of the cellular shRNA/miRNA pathway was sufficient to cause morphologic changes and even fatality in mice, it became clear that a regulated expression was important *in vivo* [48]. At the same time, shRNA sequences were discovered that are embedded in more complex RNAi transcripts, which are driven by RNA polymerase II, opening the possibility of tissue-specific expression and inclusion of a marker gene [29,49,50]. Among those, miRNA30 was first shown to mediate inhibition of cognate mRNAs with natural as well as designed miRNA sequences [51]. This system was developed further, and now allows the down-regulation of target RNAs in a tissue-specific and, by adding a doxycycline-inducible promoter, reversible manner [52–54]. 

In some situations however, as to overcome genetic redundancy or to inhibit more than one signalling pathway, it is desirable to inhibit several genes concomitantly. The miR155 precursor is embedded within the third exon of the non-coding BIC gene, and the miR155 precursor together with flanking regions is sufficient to confer the oncogenic activities of BIC/miR155 [55,56]. Based on this, a polycistronic expression vector for RNA polymerase II was developed, in which the flanking regions, which are subject to cleavage by the RNAse Drosha, are used to concatemerise sequences encoding shRNAs [25]. At least two different synthetic shRNAs can be expressed from a single transcript and coupling to a marker gene like *Gfp* is possible without compromising the efficiency of the system. We used this framework to incorporate sequences directed against *Ebf1*, *Ebf2* and *Ebf3* and observed a strong down-regulation upon ectopic expression but also of the endogenous genes, allowing us to establish a redundant role of these proteins in the support of hematopoietic stem cells [24]. As these constructs also worked in various other cellular contexts [26,27], we wanted to extend the system and to overcome the limitations of cell culture and retroviral infections.

The most striking finding of our experiments is that an RNAi construct that mediates down-regulation of cognate RNA very efficiently in cell culture does not have a biological effect *in vivo*. Several possibilities with different likelihoods can explain this discrepancy. One of our major concerns was the level of expression necessary to achieve a down-regulation *in vivo*. Originally, one of the reasons for choosing RNA polymerase III for the expression of shRNAs was the high level of expression that can be achieved. Therefore, we did not want to rely on the expression mediated by the endogenous *Rosa26* locus, which ranges from low to mid level, depending on cell type, but is not near typical RNA polymerase III or viral promotor driven expression [57,58]. Therefore, we chose the synthetic CAG promotor, which is among the strongest inducers of eukaryotic expression known [31]. We do observe a good inducibility of *Gfp* in ES cells and particularly in hematopoietic cells after *vav* ^*Cre*^ mediated activation of the transgene. In this case, the expression results in a shift of three log units in flow cytometry, and can be further enhanced to a shift over four logarithmic units by crossing the transgene to homozygosity. This expression is very likely to be higher than the levels achieved using the tet-transactivator to down-regulate p53 [52]. Therefore, and as Ebf genes are not expressed at very high levels (own unpublished data), we consider it very unlikely that too low expression of the transgene is the reason for the lack of down-regulation of Ebf proteins. The expression of the transgene at different levels allowed us to study a potential influence on Ebf protein levels closer. The analysis of cells expressing GFP not at all, or at low, middle or high levels shows that the transgene encoding is detectable at corresponding levels, which is not surprising, given that both are encoded by one mRNA. This also shows that the transgene encoding for the miRNA sequences is really present in the transgene. However, no changes in Ebf protein level can be detected, not even in the highest expressing cells, ruling out that a potential effect is masked in the bulk analysis of Gfp-positive cells and showing that the observed expression levels alone do not explain the lack of down-regulation. Furthermore, a higher expression of the transgene in homozygous transgenic animals, but no down-regulation of Ebf proteins in these mice argues against a dose-dependent effect.In addition, we conclude that expression of the transgene from the CAG promotor does not saturate the miRNA/shRNA machinery, as miRNAs control many aspects of the development of hematopoietic cells, but we do not observe any phenotypic changes [35]. 

A second possibility might be problems with the processing of a complex polycistronic mRNA *in vivo*. To generate the knock-in mice, we used exactly the same DNA construct as in the retroviral vector used for the infection of cultured murine and human cells. Therefore, since cells of the same species were used, differences in the processing machinery between cultures cells and transgenic mice seem rather unlikely. Additionally many studies show that the basic RNAi machinery is ubiquitously expressed. The second possibility is that the basic machinery is the same *in vitro* and *in vivo*, but the artificial RNAi construct generated poses more problems in its correct processing than endogenous miRNAs. Chaining of more than three shRNA sequences strongly decreases the efficiency in mediating down-regulation for each shRNA and the expression of *Gfp* is attenuated by shRNA chaining, presumably due to increased processing (data not shown). Together, this might indicate that the construct is on the border of loosing its effectiveness due to its complexity. Again, the expression of *Gfp in vitro* and *in vivo* and the down-regulation of Ebf proteins in cultured cells argues against this possibility, although we consider it more likely than the two afore mentioned possibilities.

A third general possibility is the design and efficiency of the RNAi sequences used to inhibit the Ebf proteins. The precise sequence requirements for efficient RNAi are not well understood [59], although it is known that thermodynamic asymmetry, low G/C content and a strong bias for A/U at the 5' end of the guide strand are important [60–62]. We have tested the RNAi sequences for down-regulation in cultured cells by transfection of the shRNA encoding plasmid and by retroviral infection of primary cells for efficient down-regulation of transfected and endogenous Ebf proteins [24]. In both cases however, the RNAi transcripts result from a multitude to several origins of expression (plasmids / retroviral integration sites), but not from a single genomic locus as is the case in the transgenic mice. In fact it is this aspect where the biggest differences between the *in vitro* and *in vivo* approach occur. Based on a large scale approach, it has been estimated that only 3% of all possible RNAi sequences for a particular gene elicit efficient knockdown at the level of single copies [28]. Of all the possible explanations mentioned here, we consider problems with the design and the efficiency of the RNAi sequences as single genomic copies most likely to explain the discrepancy between *in vitro* and *in vivo*, i. e. the absence of a detectable down-regulation of Ebf proteins.

In summary, we have used a new miR155-based system to explore the potential to knockdown the expression of several genes simultaneously. This approach was successfully *in vitro*, however, its direct translation *in vivo* did not result in the expected down-regulation. We think that the most likely explanation for this discrepancy lies in the efficiency of the RNAi sequences as single genomic copies. This, together with the complexity of the whole RNAi transcript, consisting of three short hairpins and *Gfp*, likely results in an overall efficiency that is too low to detect and that potentially can be compensated for. We do not think that the overall expression or differences in the endogenous basic miRNA/RNAi machinery are likely explanations. The data presented here clearly show that expression of the transgene from the CAG promoter is compatible with a normal function of the basal miRNA/RNAi machinery in hematopoietic cells, and the miR155 framework works well in the context of inducible expression from the *Rosa26* locus as shown by Gfp. The data presented here provide the foundation for future work on the simultaneous knockdown of several genes *in vivo*, as the problems concerning the efficiency of the RNAi sequences can be overcome [59].

## Experimental Procedures

### Generation of transgenic mice

Two different sequences to inhibit *Ebf1*, *Ebf2* and *Ebf3* (RNAi-a and RNAi-b) were excised together with *EmGFP* from the pcDNA6.2-GW/EmGFP vector (Invitrogen, [24]), and inserted into the *Xba*I site of the pRTS targeting vector (kindly provided by D. Calado, Immune Disease Institute Inc., Boston, MA). Mouse ES cells derived from C57Bl/6 x 129S6/SvEvTac-F1 strains (IDG3.2) were electroporated with this construct and colonies were isolated in the presence of 170 µg/ml G418 (Gibco). DNA extraction and Southern Blots were performed as described [63]. The correct insertion of the targeting sequences was analysed using an external 550 bp *5'* probe and an internal *Gfp* probe of 723 bp. The 5' probe was cut from the p*Rosa26*-5-pBS KS plasmid using *EcoR*I and *Pac*I, the *Gfp* probe was isolated from the pcDNA6.2-GW/EmGFP vector using *Dra*I. Hybridisation was carried out for 16 hours at 65°C in Church buffer (0.4 M Na_2_HPO_4_), membranes were washed 3 x 10 min at 58°C in wash buffer (0.2 x SSC, 0.5% SDS) and bands were detected using a phospoimager (Fuji Bas 1000). Two correctly targeted clones representing RNA-a and RNA-b constructs were injected into C57BL/6 blastocysts that were implanted into pseudopregnant females. Chimeric mice were bred to obtain germ-line transmission. Two male chimera (80%) corresponding to the RNA-a clone were selected to get heterozygous mice. Mutant mice were backcrossed with C57Bl/6 wild-type mice for more than 6 generations. All experiments involving animals were designed in agreement with the stipulations of the San Raffaele Institutional Animal Care and Use Committee, the Helmholtz Zentrum München and the Regierung von Oberbayern. The protocol and all experiments involving animals was approved by the ethics committees of the afore mentioned institutions. All efforts were made to minimise animal suffering.

### Genotyping

For genotyping of mice tails were digested for at least 1 h in lysis buffer [FirePol buffer B (Solis BioDyne), 1.25 mM MgCl_2_, 50 µg/ml proteinase K] at 55 °C and inactivated for 15 min at 95 °C. PCR was carried out with genomic DNA; 20 pmol of each primer were used, for the miRNA line (*5'-Rosa* fwd 5-´GAGTTCTCTGCTGCCTCCTG-3´, *CAG* rev 5´-TGAACTAATGACCCCGTAATTG-3´, *3'Rosa* rev 5´-AGGAAAGGGAAAATGCCAAT-3´ using FirePol (Solis BioDyne) under these conditions: 31 cycles of 94 °C for 45 sec, 60 °C for 45 sec, 72 °C for 1 min to amplify products of 600 bp for wild-type and 290 bp for the mutant allele; for *R26R* ^*YFP*^ line (R523 5’-GGAGCGGGAGAAATGGATAT-3’, R26F2: 5’-AAAGTCGCTCTGAGTTGTTAT-3’, oIMR 316 5’ AAGACCGCGAAGAGTTTGTC-3’) under these conditions: 35 cycles of 94 °C for 30 sec, 58 °C for 1 min, 72 °C for 1 min to amplify products of 600 bp for wild-type and 320 bp for the knock-in allele; for *Ebf2* ^*GFPiCre*^ line (F 5’-ATGGTGCCAAGGATGACTC-3´, R 5’-CCTCGAGCAGCCTCACCA-3´) under these conditions: 30 cycles of 94 °C for 30 sec, 58 °C for 30 sec, 72 °C for 30 sec to amplify a 200 bp product corresponding to the transgene.

### Cell-culture, Transfections, Plasmids

HEK293T cells were kept under standard conditions in DMEM medium (+ 10% FCS, 1% Pen/Str/Glu; Gibco), and transfected using polyethylenimine (Gibco) according to standard protocols. For ectopic expression Ebf1, Ebf2 and Ebf3 were cloned into the pcDNA3.1 vector (Invitrogen) in frame with an N-terminal Flag-tag (Sigma).

### Flow cytometry, Antibodies

For flow cytometry single cell suspensions were prepared by trypsinising ES cells or by aspirating bone marrow according to standard procedures. Isolated cells were blocked for unspecific binding using CD16/32 and incubated with either biotinylated or directly fluorochrome-conjugated antibodies. Cells were measured or sorted using either an LSRFortessa or a FACSAriaIII (BD Biosciences) and data were analysed using FACSDiva (Becton Dickinson GmbH) and FlowJo 9.3 (Treestar Inc.) software. For depletion of dead cells propidium iodide was used. Antibodies directed against the following markers were obtained from BD Biosciences: B220 (RA3-6B2) and CD43 (RM4-5), BP-1 (6C3), CD24 (30-F1), CD16/32 (=FcγRII/III; 2.4G2).

### Isolation of RNA and Quantitative RT-PCR

mRNA was isolated using peqGOLD TriFast^TM^ (Peqlab) according to manufacturer’s instructions, cDNA was synthesised using SuperScript II reverse transcriptase (Invitrogen) and the oligo-dT primer (Roche). qPCR reactions were performed in duplicate with SYBR Green I Master in a LightCycler® 480II (Roche) with standard conditions: 95 °C for 10 min followed by 45 cycles of 95 °C for 10 s, 65 °C for 10 s and 72 °C for 10 s. Primer sequences: *Hprt* fwd 5´-tgc tgg tga aaa gga cct ctc g-3´, rev 5´-tct ggg gac gca gca act ga-3´; β-Actin fwd 5'-tgt ggt ggt gaa gct gta gc-3', rev 5'-gac gac atg gag aag atc tgg-3'; *Ebf1* fwd 5´-ggg gac agt gca gat ggt aa-3´, rev 5´-caa ctc act cca gac cag ca-3´; *Gfp* fwd 5'-acc tac ggc gtg cag tgc ttc agc-3', rev 5'-gtc ctc gat gtt gtg gcg gat ctt g-3'; *miRNA*-fwd: 5'-ggc atg gac gag ctg tac aa-3', *miRNA*-rev3: ctc tag atc aac cac ttt gt-3'. Three independent measurements were performed for each qPCR analysis, error bars represent the standard deviation of the mean. The comparative CT method was used to calculate the expression levels of RNA transcripts, and the quantified individual RNA expression levels were normalised for the respective *β-actin* expression levels, unless stated otherwise. Since we measured the relative RNA expression levels, the indicated expression levels were set as 1 or 100.

### Histological procedures

Postnatal mice were anaesthetised with Avertin (Sigma, St Louis, MO, USA) and perfused with 0.9% NaCl followed by 4% paraformaldehyde (PFA). Tissues were fixed with 4% PFA, cryoprotected in 30% sucrose overnight, embedded in OCT (Bioptica), sectioned on a cryotome (18 μm). Cryosections were immunostained with the following antibodies: rabbit or mouse anti-calbindin (1:1000, Swant, Bellinzona, Switzerland); rabbit anti-GFP (1:700; Invitrogen); chicken anti-GFP (1:500; Abcam, Cambridge, UK).

### Statistics

P-values were determined with the Student’s two-tailed t-test for independent samples, assuming equal variances on all experimental datasets.

### Immunoblotting, Antibodies

Protein isolation and immunoblotting was carried out according to standard procedures, antibodies used were α-panEbf [24], α-β-Actin (AC-74, Sigma), α-Flag (M2, Sigma), The secondary α-mouse-IgG^.^HRP antibodies were obtained from Sigma, the secondary α-rat-IgG + IgM^.^HRP antibody was purchased from Jackson Immuno Research. Bands in western blot experiments were quantified using the open source software ImageJ (http://rsb.info.nih.gov/ij/; W. S. Rasband, NIH, National Institutes of Health, Bethesda, MD).

## Supporting Information

Figure S1 **Schematic overview of the DNA sequences used for RNAi.** (A) The structure of the transcripts of Ebf1, Ebf2 and Ebf3 is depicted including 5'- and 3'-UTR. Protein domains encoded by the transcripts are indicated above (HLH = helix-loop-helix domain) and the beginning and end of the coding sequence are given underneath in base pairs relative to the start site. The sequences used to inhibit the individual members and their positions are plotted against the transcripts. Two sequences are indicated for each gene, as two different RNAi constructs have been generated, and their identifying numbers are given underneath. (B) Schematic representation of the organisation of the RNAi used for the transgene. Grey boxes represent the flanking region from miR155, and the numbers within the loops indicate the Ebf gene against which the sequences at this position are directed. The composition of the two constructs used for RNAi is given below.
(TIF)

Figure S2 **Down-regulation of Ebf1, Ebf2 and Ebf3 by a single RNAi construct.** To analyse the efficiency and specificity of bioinformatically predicted sequences to down-regulate the expression of Ebf1, Ebf2 and Ebf3, HEK293T cells were transfected with expression plasmids encoding the individual Ebf proteins together with an N-terminal Flag tag. Expression vectors containing the shRNA sequences either alone or in combination were co-transfected, and 48 h after transfection cells were harvested and analysed by Western blot. α-Flag antibody was used to detect Ebf expression levels, and α-actin as loading control. Mock is referring to a co-transfection of the empty parental vector (pcDNA6.2-GW/EmGFP), no RNAi leaves out the shRNA containing vectors.
(TIF)

Figure S3 **Analysis of B cell fractions A - C in *Rosa26*^*RNAi*^ transgenic mice.** Single cell suspensions from bone marrow of mice with the indicated genotypes were analysed for the percentage of early B cell fractions. Cells were stained with propidium iodide, B220, CD43, and gated for FSC/SSC and as PI negative, and B220 CD43 double positive. The gating window and the percentage of cells are shown. Cells were further stained for BP-1 and HSA/CD24, and analysed for fractions A -C in detail (Figure 4). Representative examples of the indicated genotypes are shown. The statistical analysis of fraction A - C in Figure 4B is referring to this setting.
(TIF)
