# Correction: An RNAi-Based Approach to Down-Regulate a Gene Family *In Vivo*

**DOI:** 10.1371/annotation/c8b2e360-b78a-4c2f-a1a3-c53325f18211

**Published:** 2013-12-19

**Authors:** Jeehee Kim, Aurora Badaloni, Torsten Willert, Ursula Zimber-Strobl, Ralf Kühn, Wolfgang Wurst, Matthias Kieslinger

This article was republished on December 19, 2014 because of incorrect figures. Please download this article again to view the figures. 

