# Supplementary figures and images for: An RNAi-Based Approach to Down-Regulate a Gene Family In Vivo
Source: PLoS One. 2013 Nov 12;8(11):e80312. doi: 10.1371/journal.pone.0080312 (PMC3827190; doi:10.1371/journal.pone.0080312)

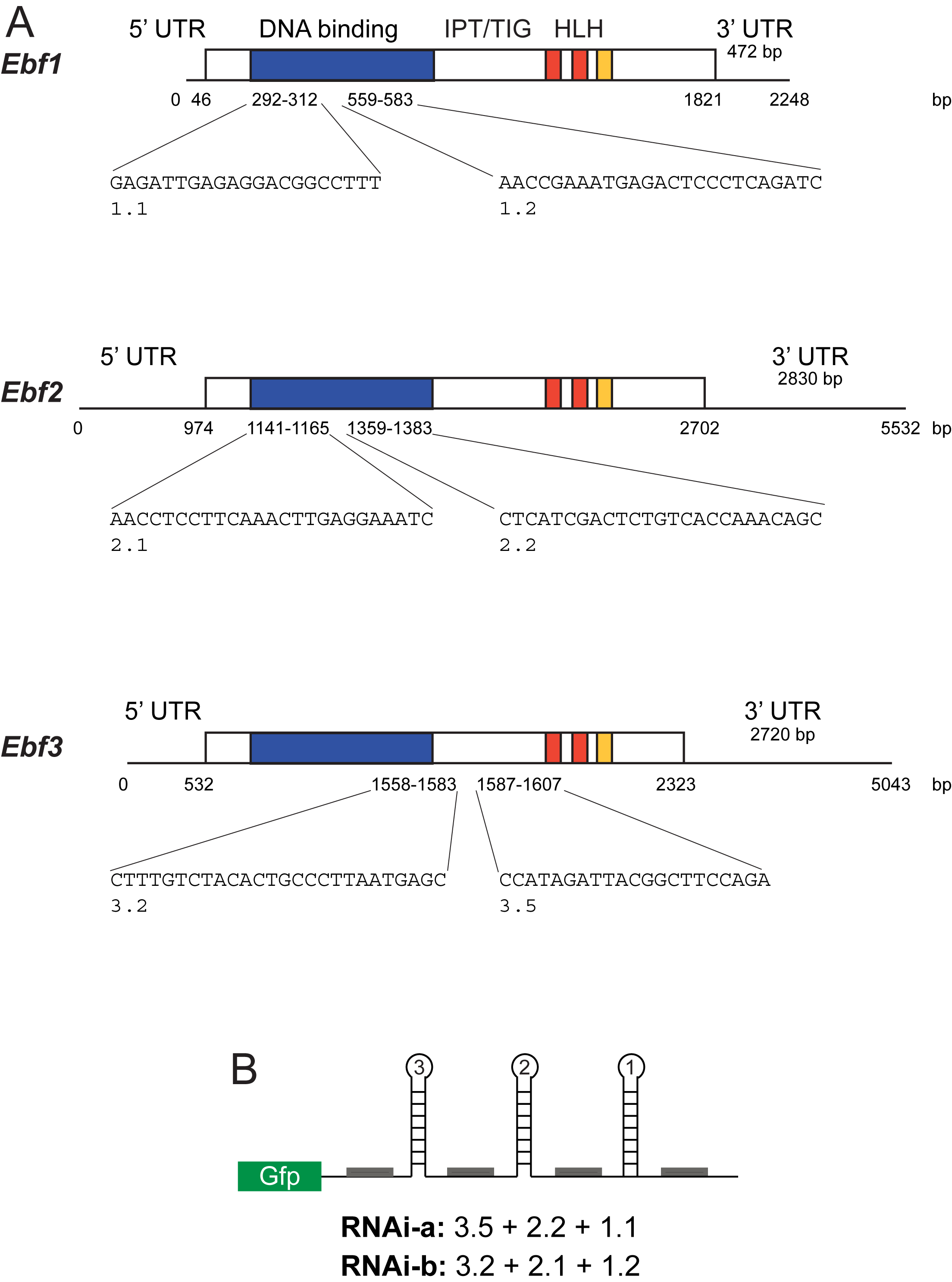

Supplement: Figure S1 — Schematic overview of the DNA sequences used for RNAi. (A) The structure of the transcripts of Ebf1, Ebf2 and Ebf3 is depicted including 5'- and 3'-UTR. Protein domains encoded by the transcripts are indicated above (HLH = helix-loop-helix domain) and the beginning and end of the coding sequence are given underneath in base pairs relative to the start site. The sequences used to inhibit the individual members and their positions are plotted against the transcripts. Two sequences are indicated for each gene, as two different RNAi constructs have been generated, and their identifying numbers are given underneath. (B) Schematic representation of the organisation of the RNAi used for the transgene. Grey boxes represent the flanking region from miR155, and the numbers within the loops indicate the Ebf gene against which the sequences at this position are directed. The composition of the two constructs used for RNAi is given below. (TIF) [file pone.0080312.s001.tif]

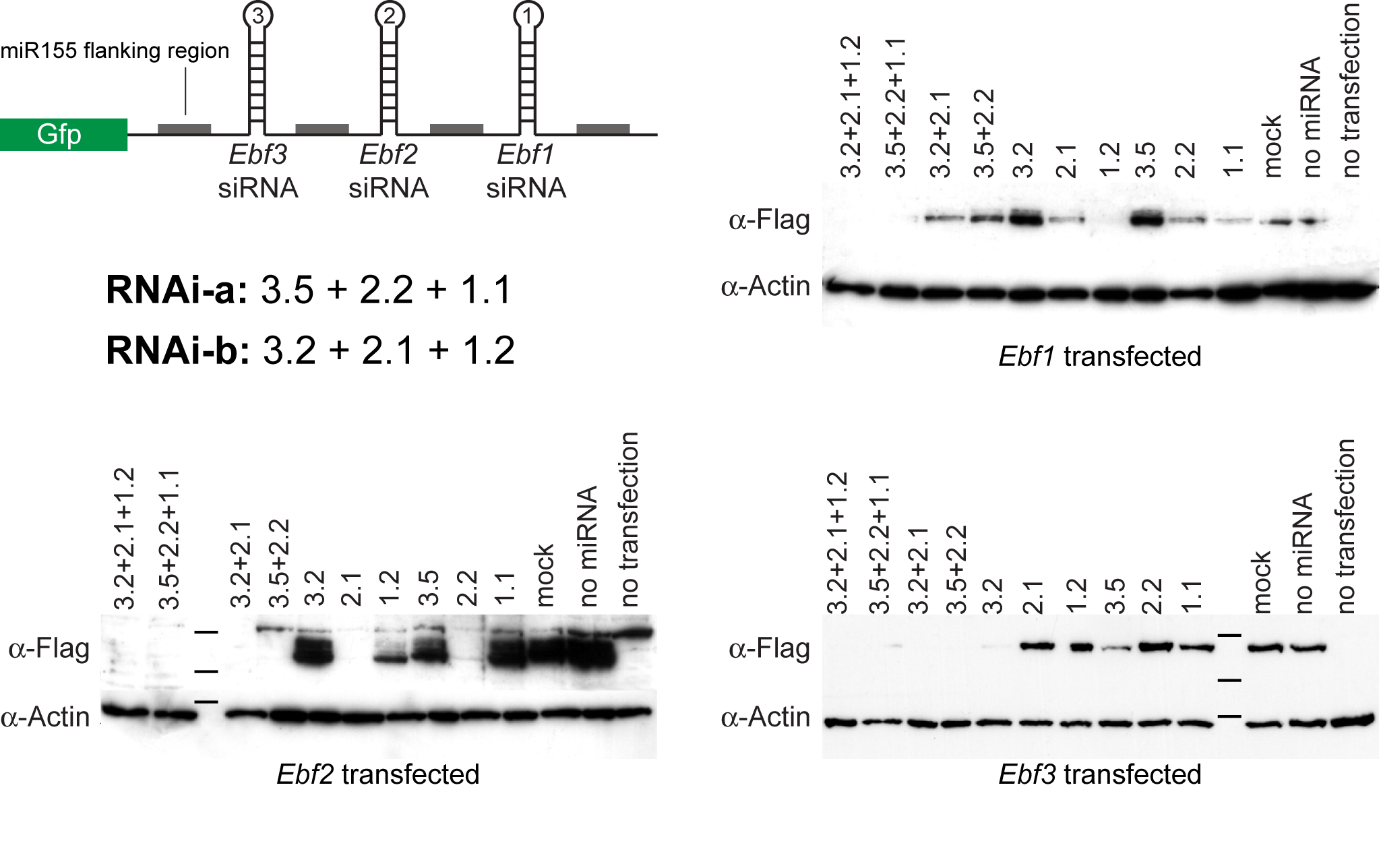

Supplement: Figure S2 — Down-regulation of Ebf1, Ebf2 and Ebf3 by a single RNAi construct. To analyse the efficiency and specificity of bioinformatically predicted sequences to down-regulate the expression of Ebf1, Ebf2 and Ebf3, HEK293T cells were transfected with expression plasmids encoding the individual Ebf proteins together with an N-terminal Flag tag. Expression vectors containing the shRNA sequences either alone or in combination were co-transfected, and 48 h after transfection cells were harvested and analysed by Western blot. α-Flag antibody was used to detect Ebf expression levels, and α-actin as loading control. Mock is referring to a co-transfection of the empty parental vector (pcDNA6.2-GW/EmGFP), no RNAi leaves out the shRNA containing vectors. (TIF) [file pone.0080312.s002.tif]

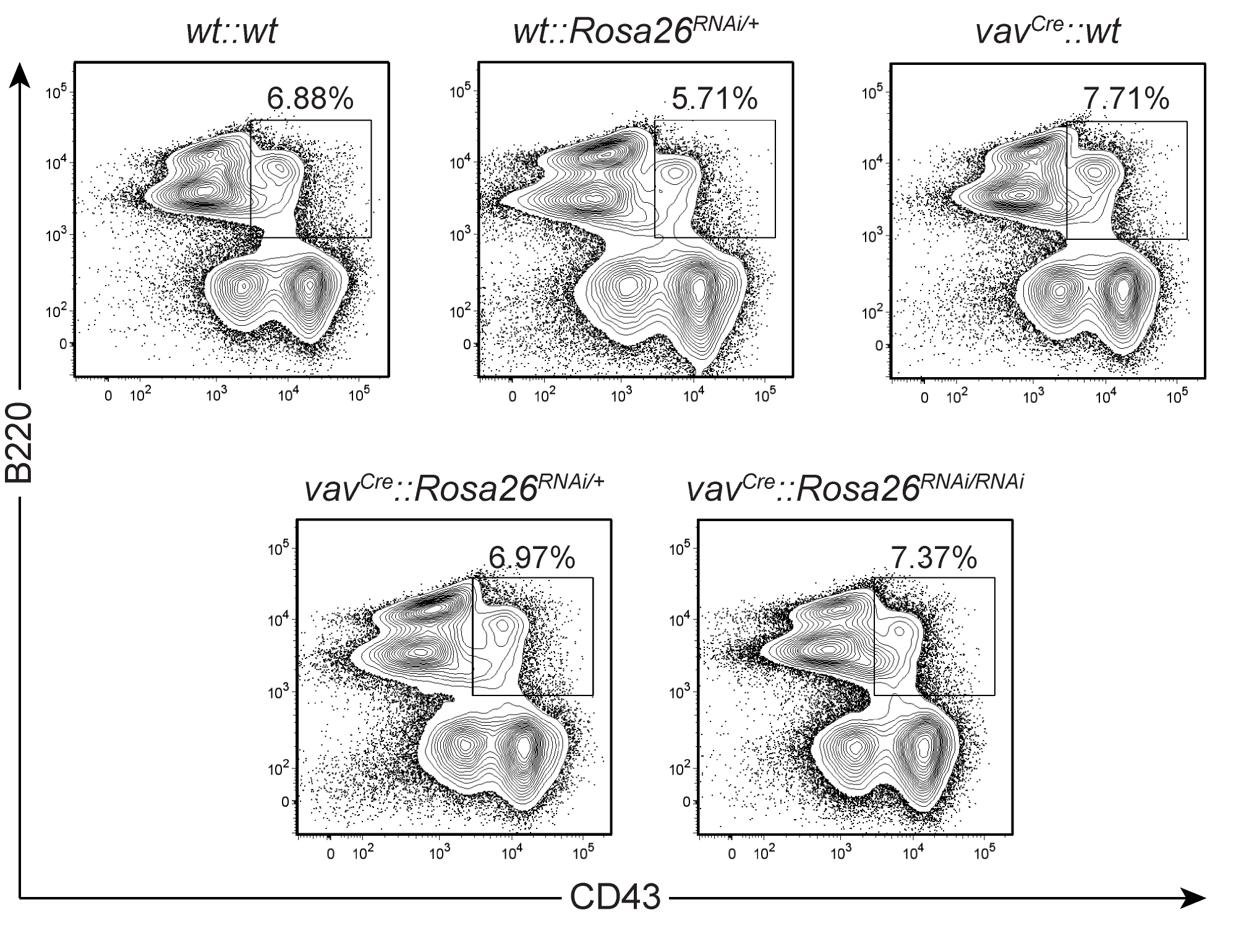

Supplement: Figure S3 — Analysis of B cell fractions A - C in Rosa26RNAi transgenic mice. Single cell suspensions from bone marrow of mice with the indicated genotypes were analysed for the percentage of early B cell fractions. Cells were stained with propidium iodide, B220, CD43, and gated for FSC/SSC and as PI negative, and B220 CD43 double positive. The gating window and the percentage of cells are shown. Cells were further stained for BP-1 and HSA/CD24, and analysed for fractions A -C in detail (Figure 4). Representative examples of the indicated genotypes are shown. The statistical analysis of fraction A - C in Figure 4B is referring to this setting. (TIF) [file pone.0080312.s003.tif]
